# Extracellular Vesicles Derived From Neural Stem Cells, Astrocytes, and Microglia as Therapeutics for Easing TBI-Induced Brain Dysfunction

**DOI:** 10.1093/stcltm/szad004

**Published:** 2023-02-27

**Authors:** Catherine Hering, Ashok K Shetty

**Affiliations:** Institute for Regenerative Medicine, Department of Cell Biology and Genetics, Texas A&M University School of Medicine, College Station, TX, USA; Institute for Regenerative Medicine, Department of Cell Biology and Genetics, Texas A&M University School of Medicine, College Station, TX, USA

**Keywords:** astrocytes, astrocyte-derived extracellular vesicles, exosomes, microglia-derived extracellular vesicles, neural stem cell-derived extracellular vesicles, neuroinflammation, traumatic brain injury

## Abstract

Extracellular vesicles (EVs) derived from neural stem cells (NSC-EVs), astrocytes (ADEVs), and microglia (MDEVs) have neuroregenerative properties. This review discusses the therapeutic efficacy of NSC-EVs, ADEVs, and MDEVs in traumatic brain injury (TBI) models. The translational value and future directions for such EV therapy are also deliberated. Studies have demonstrated that NSC-EV or ADEV therapy can mediate neuroprotective effects and improve motor and cognitive function after TBI. Furthermore, NSC-EVs or ADEVs generated after priming parental cells with growth factors or brain-injury extracts can mediate improved therapeutic benefits. However, the therapeutic effects of naïve MDEVs are yet to be tested rigorously in TBI models. Studies using activated MDEVs have reported both adverse and beneficial effects. NSC-EV, ADEV, or MDEV therapy for TBI is not ready for clinical translation. Rigorous testing of their efficacy for preventing chronic neuroinflammatory cascades and enduring motor and cognitive impairments after treatment in the acute phase of TBI, an exhaustive evaluation of their miRNA or protein cargo, and the effects of delayed EV administration post-TBI for reversing chronic neuroinflammation and enduring brain impairments, are needed. Moreover, the most beneficial route of administration for targeting EVs into different neural cells in the brain after TBI and the efficacy of well-characterized EVs from NSCs, astrocytes, or microglia derived from human pluripotent stem cells need to be evaluated. EV isolation methods for generating clinical-grade EVs must also be developed. Overall, NSC-EVs and ADEVs promise to mitigate TBI-induced brain dysfunction, but additional preclinical studies are needed before their clinical translation.

## Graphical Abstract

**Graphical Abstract:**
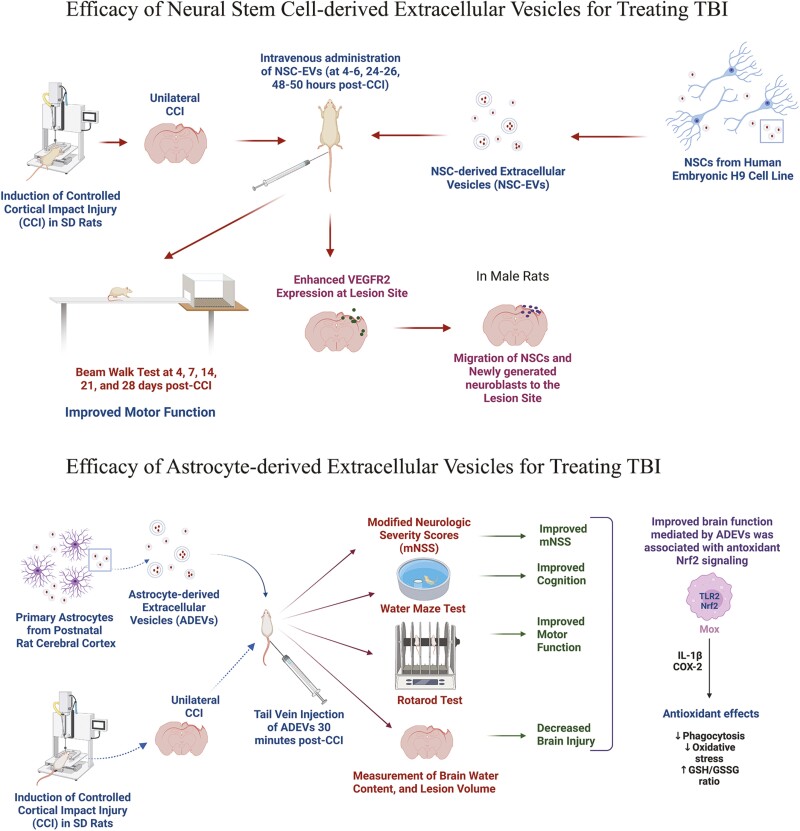

**Significance Statement**

Neural stem cell-derived extracellular vesicles (NSC-EVs) or astrocyte-derived EVs (ADEVs) can mediate neuroprotective effects and improve motor and cognitive function in models of traumatic brain injury (TBI). Furthermore, such EVs generated after priming parental cells with growth factors or brain-injury extracts can mediate improved therapeutic benefits via a specific miRNA or protein in their cargo. However, NSC-EV or ADEV therapy for TBI is not ready for clinical translation. Additional studies are needed, including testing their efficacy for preventing neuroinflammatory cascades and enduring cognitive dysfunction and examining the beneficial effects of delayed EV treatment after TBI.

## Introduction

Traumatic brain injury (TBI), neurological damage resulting from trauma, is a leading cause of disability and death worldwide, with approximately 50 million TBI cases occurring annually, creating a global financial burden of $70 billion and making TBI a major health problem.^1-6^ TBI is classified as either primary or secondary. The primary injury involves brain tissue loss from the initial insult, whereas the secondary injury is a response to the initial penetrating or closed head injury, which includes complex pathophysiological processes such as oxidative stress, neuroinflammation, altered autophagy, and neuronal death.^7-9^ The severity of TBI varies greatly depending on the secondary molecular injury cascades that could persist for years after the initial TBI, resulting in long-term cognitive and behavioral impairments, including the development of mental disorders in surviving patients.^4,10-12^ Treatment of TBI in the acute phase of injury is crucial for reducing tissue loss and improving outcomes.^13^ Currently, TBI treatment is limited to preserving nervous tissue function through surgery and pharmaceuticals to reduce oxidative stress.^14,15^ However, due to the intricate pathophysiology of secondary TBI, participating molecular targets are often ambiguous, lacking target identification, and development of effective therapies.^3,5^ Understanding the fundamental pathological mechanism of TBI is needed first to effectively treat and restore function after injury.^3^

One mechanism of neuronal injury following TBI is oxidative stress, resulting from an imbalance between reactive oxygen species (ROS) and antioxidants.^16,17^ Mitochondria produce ROS as a byproduct of oxidative phosphorylation, but mitochondrial dysfunction following TBI causes excessive ROS production, leading to oxidative damage, and apoptosis.^18-20^ Another critical pathophysiological process of secondary TBI is neuroinflammation, initiated by microglia that become activated by damage-associated molecular patterns (DAMPs) such as ROS, cellular debris, and heme released from microhemorrhages.^21,22^ Upon activation, microglia become mainly polarized to proinflammatory phenotypes, inducing adverse changes in astrocytes and exacerbating secondary injury.^21-25^ Therefore, regulating microglia activation and downstream inflammatory signaling cascades has attracted attention as a potential avenue for treating secondary TBI and preventing long-term cognitive and mood impairments.

Recently, the idea of employing neural cell-derived extracellular vesicles (EVs) to combat oxidative stress and acute neuroinflammation after TBI has emerged because of their antioxidant and anti-inflammatory cargo.^26,27^ EVs are two-layered, membrane-bound, nanosized vesicles secreted by virtually all cell types.^28-32^ EVs contain nucleic acids, proteins, and lipids and function as critical mediators of intercellular communication by transporting DNA, mRNAs, miRNAs, and proteins between cells and across the blood-brain barrier (BBB) in the nervous system.^28,33^ Broadly, there are two types of EVs: exosomes and microvesicles.^33,34^ Exosomes, ranging in size from 30 to 150 nm, originate from the endosomal pathway, while microvesicles, ranging in size from 100 to 1000 nm, bud off directly from the plasma membrane.^33,34^ Cargo loading into EVs is not random but a highly controlled process.^35,36^ EVs can be derived from various sources, including neural stem cells (NSCs), astrocytes, and microglia, and studies suggest that EVs containing large amounts of miRNAs in their cargo have potential therapeutic effects.^4,26,27,31,32,35^ Other studies have demonstrated that EVs carrying small molecules and drugs can be targeted to the brain to deliver antioxidants to neurons to reduce ROS levels.^37-40^ Such targeted delivery of small molecules via EVs could potentially treat neurological conditions and diseases, like Alzheimer’s disease and Parkinson’s disease.^3,41,42^

EVs from NSCs, astrocytes, and microglia are attractive for treating TBI due to the multiple therapeutic miRNAs and proteins they carry in their cargo.^26,27,43^ NSCs were once considered a means to replace neurons lost in the central nervous system (CNS).^4,44^ NSCs can be obtained in unlimited quantities from human embryonic or induced pluripotent stem cells, and such cells have shown promise for brain repair following injury or disease. However, widespread clinical translation of NSC therapy is yet to be realized due to potential complications linked to their use.^4,44^ These include immune rejection without significant immune suppression, low cell engraftment, the risk of developing teratomas, and the invasive approach of intracerebral transplantation.^4,44^ Furthermore, there is growing evidence that NSCs do not mediate their restorative effects via direct cell replacement but through “bystander” effects from secreted factors, including EVs.^4,45-48^ Increasingly, NSC-derived EVs (NSC-EVs) are considered an excellent alternative to NSCs as they carry NSC secretome but do not have the risks associated with NSCs, and can readily cross the BBB following intravenous or intranasal delivery.^4,26,49^ Thus, NSC-EVs offer a potential “cell-free” alternative to using NSCs that will convey therapeutic benefits without adverse side effects of NSCs.^4^

Astrocytes, a type of glia in the CNS, play an essential role in regulating CNS development, homeostasis, defense, and function.^21,50-52^ Even in physiological conditions, the EVs secreted by astrocytes (ADEVs) display neurotrophic and neuroprotective properties. However, astrocytes shed EVs carrying both neuroprotective and neuroreparative proteins/molecules in ischemic and other stressful conditions.^53-55^ ADEVs are also considered potent mediators of neural plasticity.^21,52,56,57^ Microglia are resident immune cells in the brain. Hence, naïve microglia-derived EVs (nMDEVs) likely have anti-inflammatory, neuroprotective, or neuroregenerative properties.^58-61^ On the other hand, activated MDEVs (aMDEVs) might have therapeutic or adverse effects depending on their activation state.^58-61^ Overall, due to the lack of immune rejection and their roles in neural recovery and immune regulation in the CNS, NSC-EVs, ADEVs, and nMDEVs are potentially beneficial for treating TBI and other neurodegenerative conditions involving oxidative stress and chronic neuroinflammation.^44,49^ Both NSC-EVs and ADEVs also promise to provide neuroprotection, increase hippocampal neurogenesis, and improve cognitive and mood function in disease conditions.^26,27,29,30,43,62-64^ This concise review aims to discuss highlights from studies that have tested the efficacy of NSC-EVs, ADEVs, nMDEVs, and aMDEVs in prototypes of TBI and to deliberate the translational value, limitations, and future directions for NSC-EV, ADEV or MDEV therapy for TBI.

## NSC-EVs for Treating TBI

The NSC-EV studies in brain injury models discussed in this section are listed in Table 1 with information such as the type of EVs, route of administration, the animal model employed, significant conclusions, and limitations of the study.

**Table 1.** NSC-EV, ADEV, and MDEV studies in traumatic brain injury models.

Study, type of EVs and route of administration	Animal model	Extent of injury	Major conclusions	Limitations
Sun et al. (2020)^4^ Neural stem cell-derived EVs (NSC-EVs) from human embryonic H9 cell line. Intravenous administration at 4-6 h, 24-26 h, and 48-50 h post-CCI.	A rat model of controlled cortical impact injury (CCI).	Impact at 2.17 m/s using a 3 mm tip to a depth of 2 mm with 250 ms dwell time.	NSC-EV treatment after TBI promoted neuroprotection and motor function recovery in male rats, along with increased migration of endogenous NSCs to the lesion site and increased vascular endothelial growth factor activity.	The reasons underlying sex-specific differences were not evaluated. Dose-dependent effects were not examined. The mechanisms by which NSC-EVs promoted neuroprotection and motor functional recovery were not evaluated.
Ma et al. (2019)^35^ NSC-EVs from the embryonic day 15 rat cerebral cortex NSC cultures treated with insulin-like growth factor 1 (IGF1). Intravenous administration at 1, 3-, 7-, 14-, and 28-days post-SCI.	A rat model of spinal cord injury (SCI).	SCI at T10 spinal segment using modified Allen’s weight drop apparatus.	NSC-IGF1-EV treatment improved Basso Beattie Bresnahan (BBB) locomotor scores and motor evoked potential amplitudes, reconnection of neural fasciculus, and mediated robust antiapoptotic effects. In vitro assay suggested that the neuroprotective effects of NSC-IGF1-EVs were linked to miR-219a-2-3p in their cargo.	Incorporation of NSC-EVs into neurons and glia in the injured spinal cord region was not examined. Effects of NSC-IGF1-EV therapy on SCI-induced chronic neuroinflammation and long-term motor impairments were not evaluated.
Zhang et al. (2021)^5^ Astrocyte-derived EVs (ADEVs) from cultures of postnatal rat cerebral cortex primary astrocytes. Intravenous administration at 30 minutes post-CCI.	Rat and mouse models of CCI.	Rat model: Impact at 5 m/sec to a depth of 2.5 mm with 100 ms dwell time. Impactor tip size not reported. Also, the injury parameters for the mouse model were not reported.	ADEV treatment reduced modified neurological severity score (mNSS) and improved forelimb function, motor coordination, and cognitive function with reductions in brain edema, lesion volume, neuronal atrophy, oxidative stress, and apoptosis	The mechanisms by which ADEVs mediated antioxidant effects were not assessed. The miRNAs/proteins carried by ADEVs were not characterized. The effects of ADEVs on blood-brain barrier (BBB) repair or neuroinflammation after TBI were not examined.
Long et al. (2020)^21^ ADEVs from mouse primary astrocytes treated with human traumatic brain tissue extracts.	A mouse model of CCI.	Impact at 3.5 m/s using a 5.0 mm tip to a depth of 2.0 mm with 500 msec dwell time.	ADEVs were naturally enriched with miR-873a-5p. In vitro assays revealed that miR-873a-5p can inhibit the activation of nuclear factor kappa B (NF-kB) signaling in lipopolysaccharide-treated primary microglia. Injection of miR-873a-5p agomir into the lateral ventricle after a CCI resulted in better mNSS, reduced brain damage, edema, NF-kB signaling, and M2 microglia polarization.	The efficacy of ADEVs isolated from astrocytes treated with human traumatic brain tissue extracts was not tested in the CCI model.
Chen et al. (2020)^3^ ADEVs from co-cultures of astrocytes with normal neurons or neurons damaged via air pressure. Intravenous administration at 24 hours post-TBI.	A rat model of fluid percussion injury (FPI).	Impact at 3 atmospheres (atm) pressure.	The ADEVs isolated from damaged neuron cultures displayed increased expression of gap junction alpha 1-20k (GJA1-20k). TBI rats treated with GJA1-20k expressing ADEVs displayed better preservation of brain tissues.	The efficacy of GJA1-20k containing ADEVs was not tested on cognitive, behavioral, or motor function after TBI. The mechanisms underlying neuroprotection mediated by standard ADEVs were not evaluated.
He et al. (2021)^52^ ADEVs overexpressing the nuclear transcription factor NF-κB interacting long noncoding RNA (NKILA). Intracerebral injection of NKILA-ADEVs.	A mouse model of CCI.	Impact at 3.5 m/s using a 5.0 mm tip to a depth of 2.0 mm with 500 ms dwell time.	TBI mice treated intracerebrally with NKILA-ADEVs displayed reduced mNSS, higher levels of NKILA and nucleotide-binding leucine-rich repeat-containing family member X1 (NLRX1), reduced levels of miR-195, and reduced brain tissue loss.	The potential cognitive and mood function recovery in TBI mice receiving standard or NKILA overexpressing ADEVs were not tested.
Zhao et al. (2021)^58^ Naïve microglia-derived EVs (nMDEVs) from BV2 microglial cultures and activated MDEVs (aMDEVs) from BV2 microglia co-cultured with neurons undergoing stretch injury. Intravenous administration immediately after FPI.	A mouse model of FPI.	Impact at 1.9-2.1 atm	Administration of nMDEVs promoted spine formation after TBI. However, aMDEV treatment after TBI inhibited spine density in cortical pyramidal neurons and exacerbated neurological dysfunction due to reduced expression of miR-2151 in aMDEVs. aMDEVs overexpressing miR-2151 promoted neurite outgrowth and synapse recovery after TBI by inhibiting the RhoA-Rho kinase pathway	The functional efficacy of nMDEVs after TBI was not examined.
Li et al. (2019)^61^ MDEVs enriched with miR-124-3p (miR-124 MDEVs) from cultures of BV2 microglia treated with repetitive TBI mouse brain extracts. Intravenous administration at 1 h after the first impact.	A mouse model of repetitive controlled cortical impact injury (rCCI).	Impact at 3.6 m/sec to a depth of 1.2 mm (tip size and dwell time were not reported). Repetitive impact four times at 24-h intervals.	rCCI mice receiving miR-124 MDEVs displayed reduced mNSS, improved motor performance, and enhanced spatial learning and memory.	The mechanisms underlying miR-124 MDEV-treatment mediated improved functional recovery in rCCI mice were not evaluated.
Fan et al. (2020)^65^ MDEVs generated from microglia cultured from fetal spinal cords and treated with resveratrol (RES-MDEVs). Intraperitoneal administration.	A rat model of SCI.	SCI using modified Allen’s method.	RES-MDEV treatment after SCI improved muscle tension in hind limbs and functional movements in the foot and Basso Beattie Bresnahan (BBB) scores. The beneficial effects of RES-MDEV treatment were associated with improved autophagy and reduced apoptosis.	The protein or miRNA cargos of MDEVs were not characterized. The therapeutic effects of nMDEVs were not examined.

### Proficiency of Human NSC-Derived EVs for Promoting Functional Recovery after TBI

**Figure 1.**
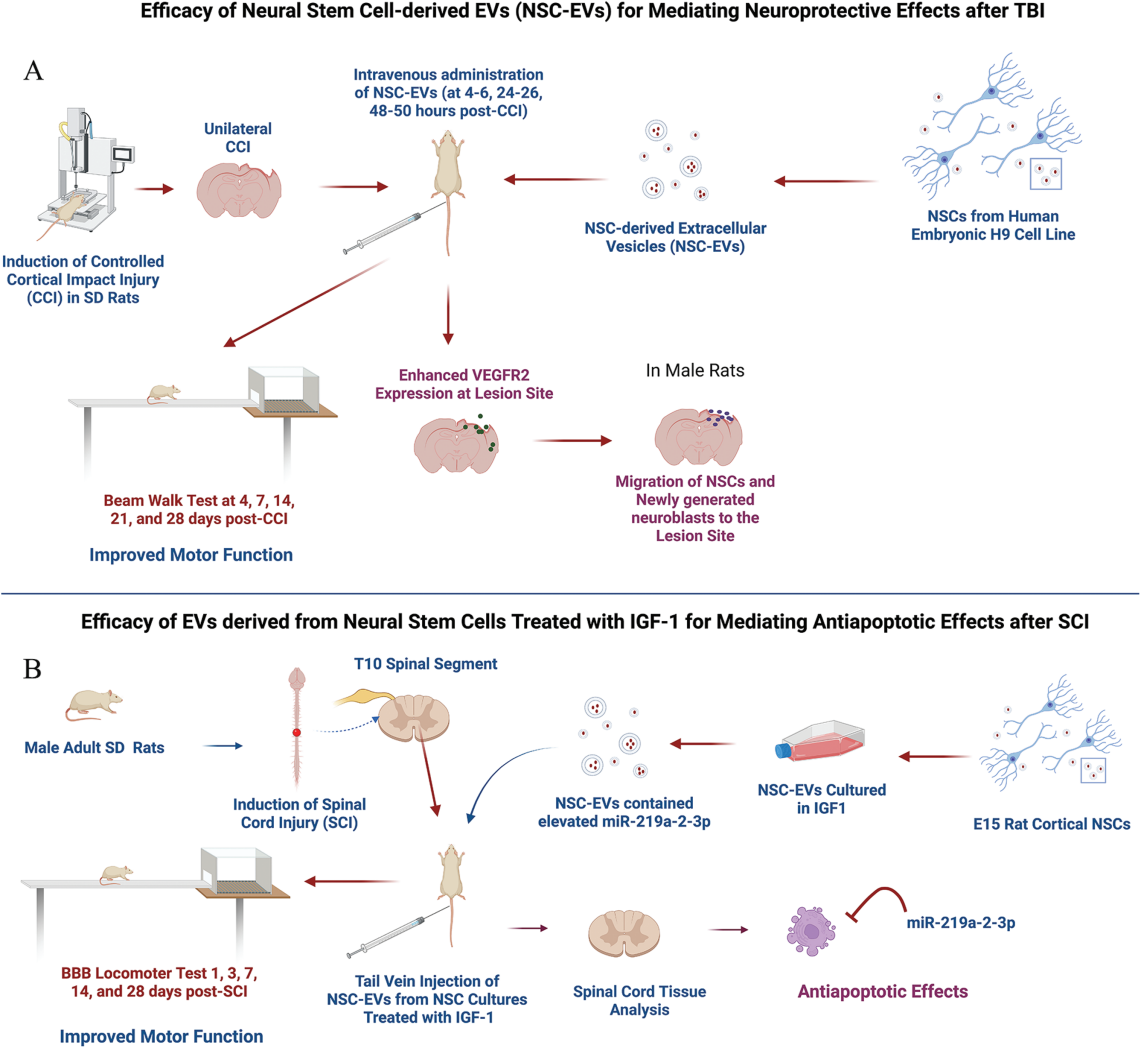
Efficacy of neural stem cell-derived extracellular vesicles (NSC-EVs) in animal models of traumatic brain injury (TBI; **A**) and spinal cord injury (SCI; **B**). A. Intravenous administration of EVs from human NSCs at 4-50 h after a controlled cortical impact injury (CCI) improved motor function, enhanced vascular endothelial growth factor receptor-2 (VEGFR2) expression in the cerebral cortex, and promoted the migration of endogenous neural stem cells and possibly newly generated neuroblasts into the lesion site. B. Intravenous administration of EVs from rat cerebral cortex NSCs treated with insulin-like growth factor-1 (IGF-1) after an SCI improved motor function and promoted neuroprotection via antiapoptotic effects. The study also demonstrated that EVs from NSCs grown in IGF-1 containing medium are naturally enriched with miR-219-2-3p, a miRNA known to mediate robust antiapoptotic effects. Sprague Dawley; T10, thoracic spinal segment 10.

Sun et al. investigated the competence of human NSC-EVs for providing neuroprotection in a model of controlled cortical impact injury (CCI).^4^ In this study, NSC-EVs isolated from human embryonic H9 cell culture media through ultrafiltration were administered intravenously to rats at 4-6 h, 24-26 h, and 48-50 h post-CCI (Fig. 1A). Assessment of fine motor coordination by measuring the number of foot faults, falls, and the final climbing distance using the beam walk test at 4, 7, 14, 21, and 28 days post-CCI suggested that NSC-EV treatment improved motor function in male rats but not in female rats. Brain tissue analysis at 4 weeks post-CCI using H&E staining showed reduced lesion area in both male and female rats. Additional analysis revealed that NSC-EV administration enhanced the migration of endogenous NSCs to the lesion site in both males and females and vascular endothelial growth factor receptor 2 (VEGFR2) expression in males without altering the overall vascular density. Overall, the study showed that NSC-EV treatment could promote neuroprotection and motor function recovery after TBI in male rats, along with increased migration of endogenous NSCs to the lesion site and increased VEGF activity (Fig. 1A).

The overall therapeutic effect was gender-specific, with female rats showing reduced beneficial effects of NSC-EVs than male rats after TBI.^4^ However, the study did not examine the reasons underlying sex-specific differences in the therapeutic properties of NSC-EVs. It could be due to increased brain damage and accelerated neuropathological changes after CCI in females due to sex hormones. Since the study did not perform the dose-response study, it remains to be addressed whether higher doses of NSC-EVs would be proficient in inducing functional recovery in females. Also, while the study noted the migration of endogenous NSCs to the lesion site and upregulation of VEGF activity, the exact mechanism by which NSC-EVs promoted neuroprotection and motor functional recovery remains elusive.^4^ Additional limitations of this study include not investigating the incorporation of intravenously administered NSC-EVs into neurons and glia and not studying the effects of NSC-EV therapy on CCI-induced chronic neuroinflammation and long-term cognitive and mood impairments.

### Competence of Rat NSC-EVs for Providing Neuroprotection After a Spinal Cord Injury

Ma et al. examined the effects of EVs secreted by rat NSCs stimulated with the insulin-like growth factor 1 (IGF1) on neuroinflammation, apoptosis, and regeneration after a spinal cord injury (SCI).^35^ The NSCs from the embryonic day 15 rat cerebral cortex were expanded as neurospheres in a standard NSC medium or an IGF1-containing NSC medium (Fig. 1B). EVs shed by NSCs were collected from the spent media via the ultracentrifugation method. Characterization of EVs isolated from neurospheres grown in the standard medium (standard EVs) and the IGF1-containing medium (IGF1-EVs) suggested that both EV types had similar morphology when examined in a transmission electron microscope. These EVs ranged in size from 30 to 300 nm, expressed EV-specific tetraspanins CD9 and CD63, and the EV-specific luminal protein, Alix. Initially, the regenerative properties of EVs were tested in PC12 cells treated with hydrogen peroxide, in which IGF1-EVs inhibited apoptosis of PC12 cells and protected axons and the overall effect of IGF1-EVs was better than standard EVs.

Next, the study employed an acute SCI model, which involved induction of injury on the spinal cord segment T10 of adult Sprague-Dawley rats using Allen’s weight drop apparatus.^35^ The animals received tail vein injections of standard or IGF1-EVs immediately after SCI, and hindlimb motor function was assessed at 1, 3-, 7-, 14-, and 28-days post-SCI using the Basso Beattie Bresnahan (BBB) locomotor rating scale and slanting board test (Fig. 1B). The BBB locomotor test scores were much better in the SCI group receiving IGF1-EVs compared to SCI alone and SCI + standard EV group. Moreover, diffusion tensor imaging implied that the reconnection of the neural fasciculus was more significant in the IGF1-EV treated groups. Furthermore, IGF1-EV treated group displayed higher motor evoked potential amplitudes, smaller injury regions, and reduced apoptosis in the injured area than other SCI groups (Fig. 1B). The robust antiapoptotic effects of IGF1-EVs could also be confirmed by the decreased expression of B-cell lymphoma-2 associated X protein (Bax), Beclin-1, and caspase-3 and increased expression of B-cell lymphoma-2 (Bcl-2) compared to other SCI groups. miRNA sequencing analysis of EVs revealed that IGF1-EVs contained higher levels of the antiapoptotic miR-219a-2-3p.^35^ Additional studies suggested that the neuroprotective effects of IGF1-EVs were linked to miR-219a-2-3p, as blockage of this miRNA led to the loss of antiapoptotic effects of IGF1-EVs on PC12 cells treated with hydrogen peroxide.

In summary, the study showed that NSCs primed with IGF-1 shed EVs with strong antiapoptotic effects due to miR-219a-2-3p in their cargo.^39^ However, since the antiapoptotic effects of miR-219-2-3p within IGF1-EVs were tested in only the cell culture model, it remains to be seen whether miR-219-2-3p can promote neuroprotection after SCI. To address this issue, future studies need to examine the effects of IGF1-EVs in SCI models with knockdown and overexpression of miR-219-2-3p. The other limitations of the study include not testing the incorporation of intravenously administered NSC-EVs into neurons and glia in the injured areas of the spinal cord and not probing the effects of NSC-EV therapy on SCI-induced chronic neuroinflammation and long-term motor impairments.

## ADEVs for Treating TBI

The ADEV studies in brain injury models conferred in this section are cataloged in Table 1 with information on the type of EVs, mode of administration, the animal prototype used, meaningful conclusions, and shortcomings of the study.

### Efficacy of Astrocyte-Derived EVs for Suppressing Oxidative Stress After a CCI

**Figure 2.**
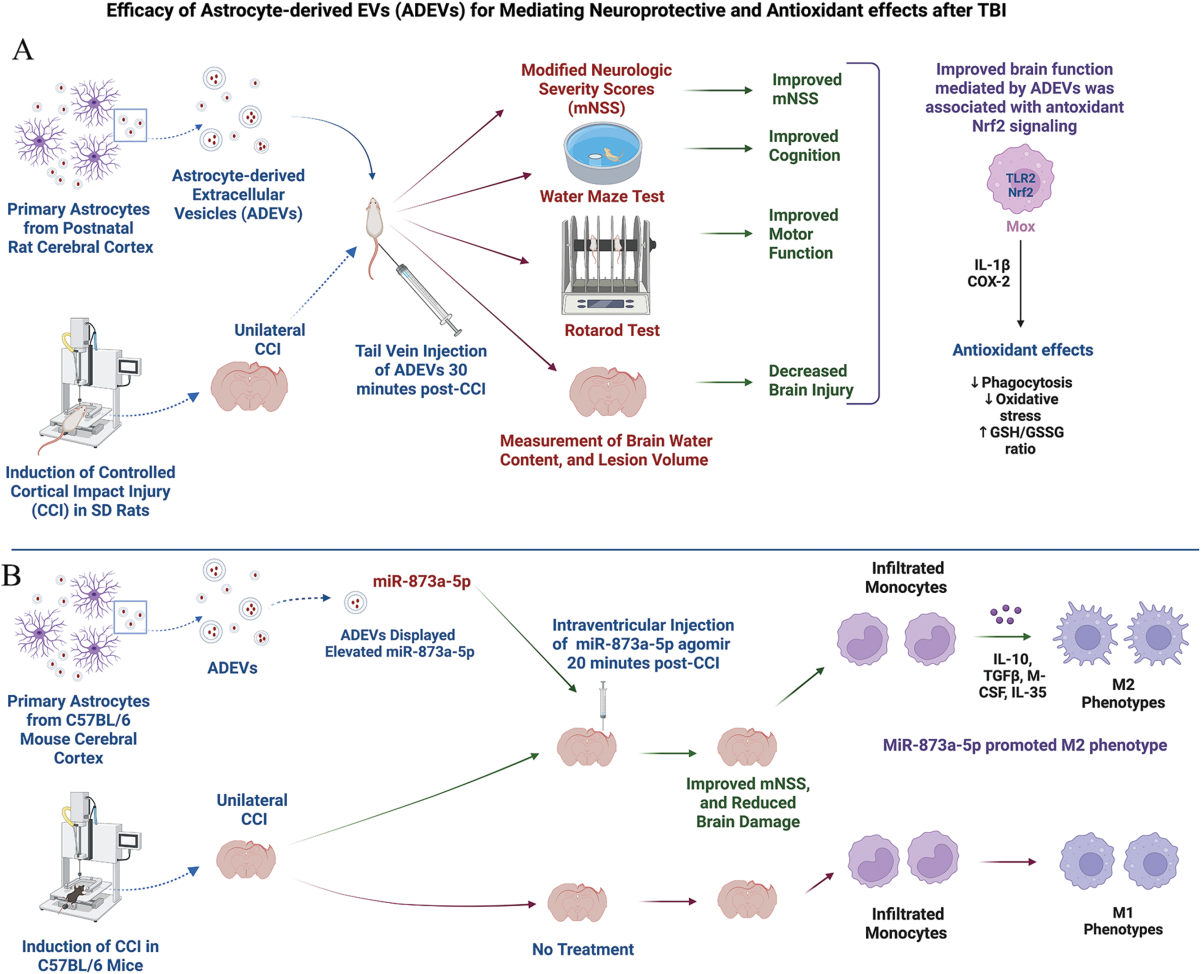
Efficacy of astrocyte-derived extracellular vesicles (ADEVs) in animal models of traumatic brain injury (TBI; **A**, **B**). A. Intravenous administration of EVs from rat primary astrocytes at 30 minutes post-controlled cortical impact injury (CCI) improved modified neurologic severity scores (mNSS), spatial learning and motor function, reduced brain water content and lesion volume, and provided neuroprotection via enhanced nuclear factor erythroid 2-related factor 2 (Nrf2) signaling. B. ADEVs from mouse primary astrocytes are naturally enriched with miR-873a-5p, a miRNA capable of modulating monocytes into M2 anti-inflammatory phenotypes. Accordingly, intraventricular injection of miR-873a-5p agomir at 20 minutes post-CCI improved mNSS, reduced brain damage, and transformed infiltrated monocytes into M2 microglial phenotypes. IL-10, interleukin-10; TGFβ, transforming growth factor-beta; M-CSF, macrophage colony stimulating factor; SD, Sprague Dawley.

Using both rat and mouse models of TBI, Zhang et al. investigated the neuroprotective effects of ADEVs and the associated mechanisms.^5^ The study utilized EVs isolated from cultures of primary astrocytes from the rat cerebral cortices of newborn Sprague-Dawley rats (Fig. 2A). The EVs, isolated from the spent media through ultracentrifugation, displayed EV-specific markers CD9, CD63, and CD81. The ADEVs were administered 30 minutes post-CCI through tail vein injection. Assessment of the severity of TBI via a modified neurological severity score (mNSS) conducted 30 minutes, 24 h, 48 h, and 7 days post-TBI revealed lower mNSS and improved forelimb function in TBI rats receiving ADEVs than the other TBI groups.^5^ Furthermore, CCI rats receiving ADEVs displayed better motor coordination in a rotarod test and improved cognitive function in a water maze test, suggesting that early ADEV administration after CCI can significantly improve both motor and cognitive function after TBI (Fig. 2A).

To determine the effects of ADEV treatment on brain pathology after CCI, the authors measured brain water content, an indicator of edema, and lesion volume for 3 consecutive days after TBI.^5^ The water content was significantly increased in the cerebral cortex and hippocampus after TBI, but ADEV treatment decreased edema, lesion volume, and neuronal atrophy (Fig. 2A). Moreover, ADEV treatment after TBI reduced ROS and hydrogen peroxide levels and increased the concentration of antioxidants such as superoxide dismutase (SOD), catalase (CAT), and glutathione (GSH) in the hippocampus. These antioxidant effects of ADEVs were associated with activation of the nuclear factor-erythroid factor 2-related factor 2 (Nrf2) signaling pathway, the master regulator of oxidative stress. Additional analysis revealed that ADEV administration after TBI increased the heme oxygenase-1 pathway and reduced apoptosis, evident from reduced levels of Bax to Bcl-2 ratio and CC-3 (TIP30) protein in the hippocampus.^5^ To confirm that ADEVs reduced oxidative stress by activating the Nrf2 signaling pathway, the authors compared the effects of ADEVs after CCI in Nrf2^+/+^ mice vis-a-vis Nrf2 knockout (KO) mice. While ADEV treatment reduced ROS and hydrogen peroxide levels with increased SOD and CAT levels in Nrf2^+/+^ mice, such effects were not seen in Nrf2-KO mice. The authors also demonstrated that ADEVs decrease apoptosis through the activation of Nrf2 (Fig. 2A). This result was evident from decreased Bax to Bcl-2 ratio and CC3 in Nrf2^+/+^ mice receiving ADEVs after TBI, compared to Nrf2-KO mice undergoing TBI displaying higher levels with or without ADEV treatment. Overall, the study demonstrated that early administration of ADEVs after TBI can reduce tissue injury and motor and cognitive deficits through potent antioxidant effects involving activation of the Nrf2 signaling pathway. However, the mechanisms by which ADEV-mediated antioxidant effects were not explored in the study, as the miRNA or protein cargo of ADEVs were not examined. Also, the effects of ADEVs were not tested on BBB repair or neuroinflammation after TBI.

### Properties and miRNA Composition of EVs Derived from Primary Astrocytes Treated with Human Traumatic Brain Tissue Extracts

Long et al. investigated ADEVs collected from C57BL/6 mouse primary astrocytes following treatment with human traumatic brain tissue extracts.^21^ The EVs were isolated from astrocyte culture media using ultracentrifugation and characterized via transmission electron microscopy (TEM) and western Blot analysis of CD9 and CD63. Through genome-wide microarray analysis, the authors identified miR-873a-5p as one of the top 5 miRNAs among ~132 miRNAs within these ADEVs. Based on the bioinformatics database, the authors identified miR-873a-5p as the likely candidate affecting nuclear factor kappa B (NF-kB) signaling and focused all further studies on this miRNA. They confirmed the upregulation of miR-873a-5p in human traumatic brain tissue samples from necrotic and edema areas. Next, they demonstrated that transfection of miR-873a-5p into primary microglia stimulated with lipopolysaccharide (LPS) can result in a decreased secretion of inducible nitric oxide synthase (iNOS), high mobility group box protein 1 (HMGB1), and proinflammatory cytokines ­interleukin-1 beta (IL-1β), tumor necrosis factor-alpha (TNF-α) and IL-6, compared to microglia challenged with LPS alone. Additional characterization also demonstrated that intervention with miR-873a-5p can inhibit the activation of NF-kB signaling in LPS-treated primary microglia.^21^

Further studies in a mouse model of CCI showed that injection of miR-873a-5p agomir into the lateral ventricle immediately after a CCI can increase miR-873a-5p levels in the cortex at 1, 3, and 7 days post-TBI (Fig. 2B). Such an increase resulted in better mNSS scores and reduced brain damage and edema at 7 days post-TBI.^21^ Additional characterization revealed that miR-873a-5p promoted M2 polarization by inhibiting M1 signature genes iNOS, CD32, IL-1β and promoting M2 genes CD206, IL-4, and arginase-1 (Fig. 2B). Immunofluorescence staining for M1 and M2 markers also confirmed such microglial polarization. Western blot analysis of myeloid differentiation primary response 88 (Myd88), phosphorylated extracellular signal-regulated kinase (p-ERK), and NF-κB p65 also suggested a reduction in the activation of the NF-κB signaling pathway. Overall, the study identified the promise of miR-873a-5p for reducing neuroinflammation after a CCI. However, these results are not congruent with a previous study showing that miR-873 released from astrocytes can promote inflammation through the A20/NF-kB pathway.^66^ Additionally, the limitation of the study is that the authors have not tested the efficacy of ADEVs isolated from astrocytes treated with human traumatic brain tissue extracts in cell culture or in vivo TBI models. Therefore, it is unknown whether stimulating primary astrocytes with traumatic brain tissue extracts is an efficient avenue for obtaining highly therapeutic ADEVs for treating conditions such as TBI.

### Proficiency of ADEVs Carrying Gap Junction Alpha 1 for Reducing Neuronal Apoptosis and Mitochondrial Function After TBI

**Figure 3.**
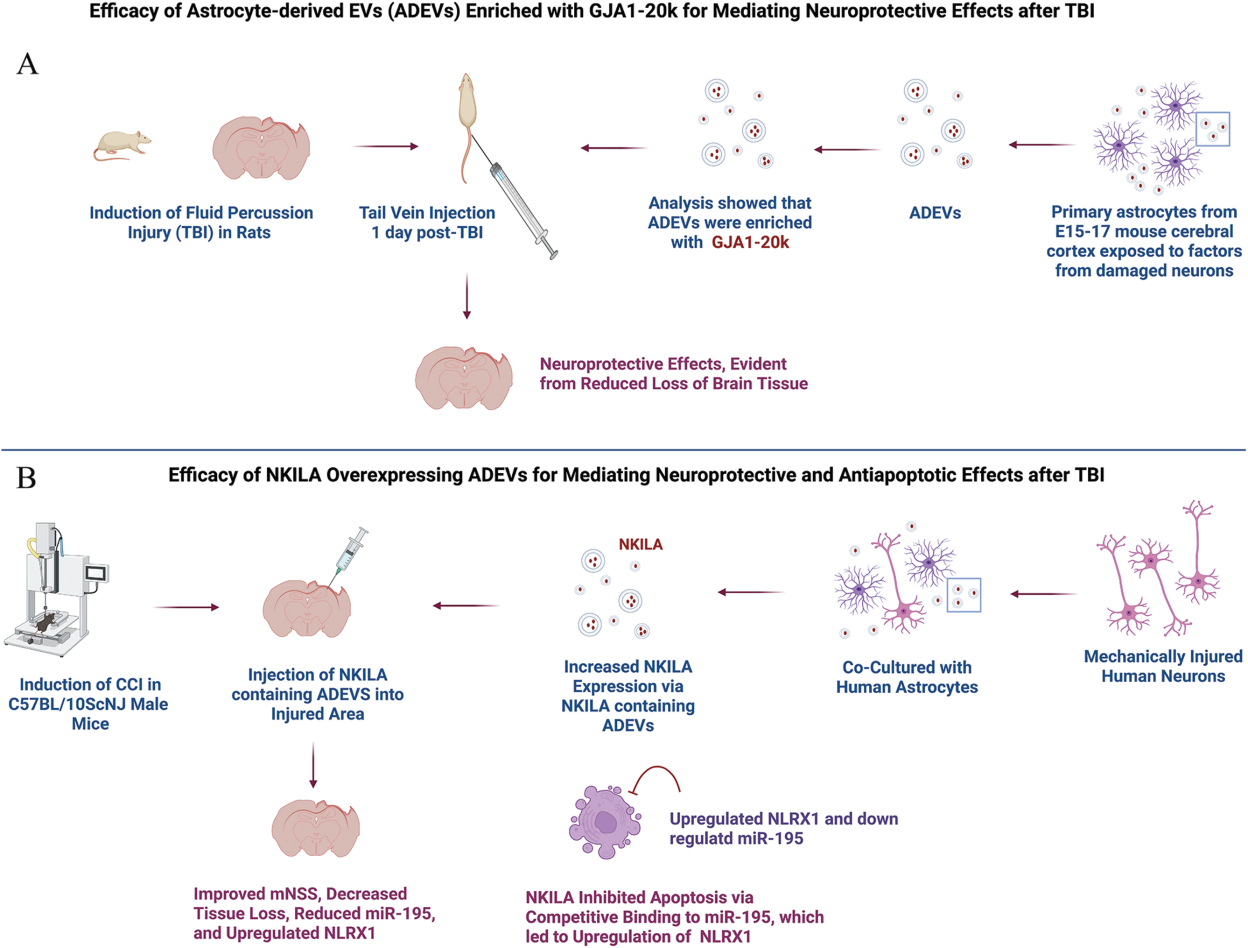
Efficacy of astrocyte-derived extracellular vesicles (ADEVs) enriched with a gap junction protein alpha GJA1-20k (**A**) or nuclear factor kappa B (NF-κB) interacting long noncoding RNA (NKILA; **B**) in animal models of traumatic brain injury (TBI). A. ADEVs generated from mouse primary astrocytes exposed to factors from damaged neurons were enriched with GJA1-20k. Intravenous administration of such ADEVs one day after TBI provided neuroprotection. B. A direct intraventricular injection of ADEVs overexpressing NKILA after a controlled cortical impact injury (CCI) improved modified neurologic severity scores (mNSS) and decreased brain tissue loss. NKILA inhibited apoptosis by binding to miR-195, which upregulated free nucleotide-binding leucine-rich repeat-containing family member X1 (NLRX1) levels.

To determine if ADEVs carrying gap junction alpha 1-20k (GJA1-20k), the mRNA of a chaperone protein maintaining mitochondrial stability, are neuroprotective after TBI, Chen et al. established a transwell culture of rat astrocytes and neurons.^3^ ADEVs were generated following co-culturing of astrocytes with normal neurons or neurons damaged via air pressure. The EVs were isolated through ultracentrifugation and characterized via TEM, NanoSight, and Western Blot analysis for CD60, CD9, and Hsp70. The ADEVs isolated from damaged neuronal cultures displayed increased expression of GJA1-20k (Fig. 3A). Next, GJA1-20k expressing ADEVs and ADEVs with GJA1-20k knock-out (KO) were tested on cultured damaged neurons. While GJA1-20k expressing ADEVs improved neuronal survival through downregulation of apoptosis, ADEVs with GJA1-20k KO failed to reduce apoptosis, evidenced by increased levels of pro-apoptotic proteins such as cytoC, Bcl-2, Bax, and caspase 3 in damaged neurons treated with GJA1-20k KO EVs. These results implied that GJA1-20k protects neurons by downregulating apoptosis. Additional investigation revealed that damaged neurons displayed reduced Cx43 phosphorylation when treated with GJA1-20k expressing ADEVs but not when treated with GJA1-20k KO EVs.^3^ Treatment of damaged neurons with GJA1-20k expressing ADEVs also restored peroxisome proliferator-activated receptor gamma co-activator 1 alpha (PGC1α) and human mitochondrial transcription factor A (mtTFA), the activators of mitochondrial biosynthesis, Tom20, important in mitochondrial internal transporter function, cytochrome c oxidase subunit 2 (mTCO2), ATP content, and lactate/pyruvate ratio. GJA1-20k expressing ADEVs also promoted recovery of mitochondrial ultrastructure and neuronal morphology. However, such effects were not seen when damaged neurons were treated with GJA1-20k KO EVs, implying that GJA1-20k is a significant neuroprotectant in ADEVs.^3^

To verify the results of the cell culture assay, the authors tested the efficacy of GJA1-20k expressing ADEVs and GJA1-20k KO ADEVs in a rat model of TBI utilizing hydraulic injury (Fig. 3A). The EVs were administered via tail vein injection at 1-day post-TBI, and brain tissues were evaluated at 1-week post-TBI.^3^ The results showed that TBI rats treated with GJA1-20k expressing ADEVs displayed better preservation of brain tissues than TBI rats receiving GJA1-20k KO ADEVs. The study identified GJA1-20k as a vital component of ADEVs mediating neuroprotection. However, this study has limitations, including no testing of cognitive, behavioral, or motor function after administering GJA1-20k containing ADEVs or GJA1-20k KO ADEVs in TBI rats to assess the functional effects of GJA1-20k. Also, the mechanisms underlying neuroprotection mediated by standard ADEVs were not evaluated.

### Ability of Astrocyte-Derived EVs Overexpressing the lncRNA NKILA for Mediating Neuroprotection and Anti-Inflammatory Effects After a CCI

He and associates investigated the effects of ADEVs overexpressing the nuclear transcription factor NF-κB interacting long noncoding RNA (NKILA) on TBI.^52^ NKILA has a binding site for miR-195, and miR-195 bound to nucleotide-binding leucine-rich repeat-containing family member X1 (NLRX1) stimulates ROS production to amplify NF-κB signaling while unbound NLRX1 inhibits it. Therefore, the authors hypothesized that ADEVs carrying NKILA promotes recovery after TBI, as binding of NKILA to miR-195 facilitates NLRX1 to inhibit NF-κB signaling. The authors first tested these EVs in an in vitro model of TBI comprising mechanically injured human neurons.^52^ Then, they tested the efficacy of these EVs for neuroprotection in a mouse model of CCI. In the cell culture model, authors found that NKILA was downregulated in injured neurons but coculturing with astrocytes increased NKILA expression in injured neurons with corresponding increases in cell viability. Further analysis revealed that NKILA from astrocytes was transferred into injured neurons via EVs, which resulted in the reduction of miR-195 and upregulation of NLRX1. These changes facilitated increased proliferation and decreased apoptosis of neurons, which was evident from the reduced expression of proapoptotic genes Bax and caspase-3. The authors also demonstrated the ability of NKILA to upregulate NLRX1 by competitively binding to miR195 and the overexpression of NKILA suppressing the apoptosis of injured neurons through upregulation of NLRX1.^52^

To test the efficacy of NKILA-overexpressing ADEVs in the mouse model of CCI, ADEVs were injected directly into the injured cortex, which reduced mNSS scores (Fig. 3B). Furthermore, brain tissue analysis confirmed reduced NKILA and NLRX1 levels, increased miR-195, and significant brain tissue loss in the TBI mice.^52^ TBI mice receiving NKILA enriched EVs displayed higher levels of NKILA and NLRX1, reduced levels of miR-195, and better brain tissue preservation (Fig. 3B). In summary, the study demonstrated that ADEVs carry NKILA, and delivering higher levels of NKILA can significantly reduce brain tissue loss after TBI. However, the functional effects of NKILA remain to be investigated as this study did not analyze the potential recovery of cognitive and mood function in TBI mice receiving standard or NKILA overexpressing ADEVs.

## MDEVs for Treating TBI

The MDEV studies in brain injury prototypes detailed in this section are listed in Table 1 with information such as the type of EVs, route of administration, the animal model used, major conclusions, and limitations of the study.

### EVs Derived From Naïve Microglia and Activated Microglia Display Contrasting Effects on Structural and Functional Recovery After TBI

Zhao et al. tested the inhibitory effects of aMDEVs on neurite outgrowth and synapse recovery after TBI.^58^ Activated microglia were induced through co-culturing of naïve microglia (BV2 primary microglia) with primary neurons isolated from embryonic day 18 mice undergoing stretch injury. aMDEVs were next isolated through ultracentrifugation and labeled with PKH26. Labeled aMDEVs (2.5 × 10^10^) were administered intravenously through tail vein injections into Thy1-green fluorescent protein (GFP) knock-in mice subjected to lateral fluid percussion injury (FPI). The study employed a moderate TBI model with the peak impact pressure controlled at 1.9-2.1 atmospheres (atm), equivalent to ~30 pounds per square inch (PSI). aMDEVs incorporated into pyramidal neurons in cortical layers V and VI, and decreased growth-associated protein-43 (GAP-43) expression in neurons in the peri-contusion region. Such changes were associated with increased foot slips in FPI mice receiving aMDEVs compared to FPI mice receiving vehicle treatment in the beam walking test.^58^ Moreover, spine density in apical dendrites of pyramidal neurons was decreased significantly in the peri-contusion region of mice receiving aMDEVs. In contrast, intravenous administration of nMDEVs isolated from naïve BV2 primary microglial cultures significantly increased apical dendrite spine density.

Cell culture studies revealed that adverse effects of aMDEVs were due to reduced expression of miR-5121, as overexpression of miR-5121 in these EVs increased the expression of GAP-43 and other synaptic proteins and enhanced dendritic complexity in cultured neurons. Besides, administration of aMDEVs overexpressing miR-5121 into FPI mice enhanced GAP-43 expression, apical dendrite spine density in the peri-contusion region, and improved motor coordination. Additional investigation revealed that MDEVs overexpressing miR-5121 also decreased the upstream activator of the RhoA-Rho kinase pathway and its downstream effector RhoA-GTP in the peri-contusion region.^58^ Overall, the study demonstrated that the administration of nMDEVs promotes spine formation after TBI. In contrast, aMDEV treatment inhibits spine density in cortical pyramidal neurons and exacerbates neurological dysfunction after TBI due to reduced expression of miR-2151 in aMDEVs. The study also uncovered the beneficial effects of aMDEVs overexpressing miR-2151 in promoting neurite outgrowth and synapse recovery after a moderate TBI by inhibiting the RhoA-Rho kinase pathway. The limitations of this study include no testing of nMDEVs on functional recovery after TBI and no examination of the effects of a MDEVs overexpressing miR-5121 on cognitive function.

### MDEVs Enriched With miR-124-3p Promote Functional Recovery After Repetitive TBI

Li et al. tested the efficacy of MDEVs enriched with miR-124-3p (miR-124 MDEVs) in a mouse model of repetitive CCI (rCCI) that employed 4 impacts at 24-h intervals, at a velocity of 3.6 m/s and a deformation depth of 1.2 mm.^61^ miR-124 MDEVs were isolated from cultures of BV2 microglia treated with repetitive TBI mouse brain extracts. In an in vitro scratch neuronal injury model, administration of miR-124 MDEVs promoted neuroprotective effects by regulating the exaggerated upregulation of autophagy and apoptotic markers typically seen in injured neurons by targeting 3ʹ UTR of family-interacting protein of 200 kDa (FIP200) mRNA. While autophagy is beneficial in physiological conditions because of its ability to maintain cellular homeostasis by eliminating damaged cellular components, overly upregulated autophagy after TBI can contribute to increased neurodegeneration.^61^ Indeed, studies have shown that inhibition of excessive autophagy after TBI can improve neurological outcomes.^67,68^ Therefore, in this study, the authors tested the effects of intravenous administration of miR-124 MDEVs in rCCI mice. Following treatment with miR-124 MDEVs, rCCI mice displayed reduced mNSS scores, improved motor performance in the rotarod test, and enhanced spatial learning and spatial memory retrieval ability compared to the rCCI alone group.^61^ The results demonstrated the efficacy of miR-124 MDEVs for mediating functional recovery after rCCI. However, the authors did not test the effects of miR-124 MDEVs on autophagy in the brain, which is a limitation of the study. Therefore, mechanisms underlying miR-124 MDEV-treatment mediated improved functional recovery in rCCI mice remain to be determined, though suppression of autophagy is one of the likely mechanisms.

### Resveratrol-Loaded MDEVs Promote Motor Recovery After SCI by Activating Autophagy and Inhibiting Apoptosis

Fan et al. investigated the effects of the administration of MDEVs generated from microglia cultured from fetal spinal cords and treated with the antioxidant and anti-inflammatory compound resveratrol (RES-MDEVs) in a rat model of SCI that employed Allen’s method.^65^ The RES-MDEVs were isolated through ultracentrifugation and were administered 14 days post-SCI. The authors demonstrated that resveratrol in RES-MDEVs was more stable than free resveratrol, with increased concentrations detected in the lung, liver, brain, and spinal cord in vivo. RES-MDEV treatment after SCI improved muscle tension in hind limbs and functional movements in the foot, compared to the SCI alone group or SCI group receiving free resveratrol. The SCI rats receiving RES-MDEV treatment also displayed better BBB scores. The beneficial effects of RES-MDEV treatment were associated with the upregulation of autophagy-related proteins microtubule-associated protein light chain 3 beta (LC3B) and beclin-1, and inhibition of apoptosis-related protein cleaved caspase-3. The results suggested that RES-MDEVs can improve motor recovery in rats after SCI through increased autophagy and decreased apoptosis. However, the study has several limitations. Apart from resveratrol analysis, the study did not characterize the protein or miRNA cargo of MDEVs. Also, the study did not employ a naïve MDEV treatment group. Hence, it is unclear whether the effects were due to the delivery of resveratrol by MDEVs or the therapeutic cargo of MDEVs.

## Overall Conclusions and Future Perspectives

NSC-EVs and ADEVs have the potential to treat TBI. Several preclinical studies show that NSC-EV administration after a CCI or SCI can mediate neuroprotective effects and promote motor recovery. Also, EVs from NSCs primed with IGF1 have better antiapoptotic effects because of specific miRNAs in their cargo. Furthermore, early intervention with ADEVs after TBI appears promising for promoting motor and cognitive recovery by antioxidant effects via enhanced Nrf2 signaling. The ADEVs generated from primary astrocytes treated with traumatic brain tissue extracts appear to be loaded with a specific miRNA that can transform activated microglia into non-inflammatory or anti-inflammatory M2 phenotypes by inhibiting NF-κB signaling. Additionally, ADEVs generated from astrocytes cultured in the presence of injured neuron factors carry high levels of GJA1-20k, having robust neuroprotective activity. Another study has demonstrated that ADEVs overexpressing NKILA could further enhance the antioxidant, NF-kB signaling inhibition, and antiapoptotic effects of ADEVs. Concerning MDEVs, the therapeutic effects of nMDEVs are yet to be tested rigorously in TBI models. On the other hand, studies using aMDEVs have reported both adverse and beneficial effects. One study demonstrated that aMDEV treatment inhibits spine density in cortical pyramidal neurons and exacerbates neurological dysfunction after TBI due to reduced expression of miR-2151 in aMDEVs.^58^ Whereas, another study showed that aMDEVs isolated from cultures of BV2 microglia treated with repetitive TBI mouse brain extracts could improve functional recovery in a model of rCCI.^61^ Thus, therapeutic or adverse effects of aMDEVs likely depend on the microglial activation state. Considering these, it may be necessary to utilize MDEVs shed from microglia displaying either non-inflammatory or anti-inflammatory properties.

Nonetheless, several issues remain regarding the use of NSC-EVs, ADEVs, or MDEVs as “biologic” for treating TBI in the future. First, rigorous and long-term testing of motor, cognitive, and mood function after NSC-EV, ADEV, or MDEV administration after TBI has not been performed. Several studies have employed only mNSS to gauge therapeutic effects. Second, most studies have mainly tested the neuroprotective, anti-apoptotic, and anti-inflammatory effects of such EVs in the acute phase of TBI. Since secondary changes after TBI evolve over weeks and months after TBI,^69-71^ the effect of early NSC-EV, ADEV, or MDEV treatment after TBI needs to be examined on chronic neuroinflammatory cascades associated with long-term cognitive and mood impairments. Third, the efficacy of delayed interventions using NSC-EVs, ADEVs, or MDEVs after TBI has not been ascertained. Such studies are vital to determine whether neuroinflammatory cascades and enduring cognitive and mood impairments could be reversed through single or intermittent administration of NSC-EVs, ADEVs, or MDEVs commencing days or weeks after experiencing TBI. Fourth, the best route of EV administration for treating TBI has not been evaluated. Most studies employed tail vein injections in rodent models but have not quantified the fraction of EVs that permeate the brain or get incorporated into neurons and glia. Since intravenous administration leads to the entry of EVs into the systemic circulation, the amount of EVs entering the brain is likely minimal. Therefore, testing the efficacy of intranasal administration in TBI models will be necessary as such administration in other models has shown quick entry of EVs into virtually all regions of the brain as well as their incorporation into neurons, microglia, and astrocytes.^26,30,72^

Furthermore, while some studies have identified one or more miRNA or protein cargo having neuroprotective, antioxidant, or anti-inflammatory effects, an exhaustive evaluation of miRNA or protein cargo of NSC-EVs, ADEVs or MDEVs has not been conducted. Such characterization is vital as NSCs, astrocytes, and microglia grown in different culture conditions could carry some adverse miRNAs and proteins in addition to beneficial molecules. Therefore, evaluating the function of highly enriched miRNAs and proteins within NSC-EVs, ADEVs, and MDEVs through gain of function and loss of function studies in cell culture assays will be crucial. Since secondary changes contribute to illness at extended time points after TBI, a thorough analysis of NSC-EV, ADEV, or MDEV cargo will be vital to understand the potential mechanisms by which EVs mediate recovery of function and further improve their therapeutic efficacy. Moreover, most studies have employed NSC-EVs, ADEVs, or MDEVs from rat or mouse NSCs, astrocytes, and microglia. Hence, testing the efficacy of well-characterized EVs from NSCs, astrocytes, or microglia derived from human embryonic or induced pluripotent stem cells^26^ in penetrating TBI and closed head injury models will be needed in the future to translate EV therapy for both mild and moderate TBIs in the clinic. Also, EV isolation methods compatible with generating clinical grade EVs as per FDA guidelines must be developed before clinical translation of NSC-EVs, ADEVs, or MDEVs is considered for treating TBIs in the clinic. In conclusion, NSC-EVs and ADEVs have promise for mitigating TBI-induced brain dysfunction. However, significant additional preclinical studies in various models of TBI using NSC-EVs and ADEVs that have undergone thorough characterization of their cargo as well as therapeutic and adverse properties are needed prior to clinical translation.

## Data Availability

All data needed to evaluate the conclusions of this concise review are present in the article.

## References

[1] Johnson WD , Griswold DP. Traumatic brain injury: a global challenge. Lancet Neurol. 2017;16(12):949-950. 10.1016/S1474-4422(17)30362-9 29122521

[2] Maas AIR , Menon DK, Adelson PD, et al; InTBIR Participants and Investigators. Traumatic brain injury: integrated approaches to improve prevention, clinical care, and research. Lancet Neurol. 2017;16(12):987-1048. 10.1016/S1474-4422(17)30371-X 29122524

[3] Chen W , Zheng P, Hong T, et al. Astrocytes-derived exosomes induce neuronal recovery after traumatic brain injury via delivering gap junction alpha 1-20 k. J Tissue Eng Regen Med. 2020;14(3):412-423. 10.1002/term.3002 31826322

[4] Sun MK , Passaro AP, Latchoumane CF, et al. Extracellular vesicles mediate neuroprotection and functional recovery after traumatic brain injury. J Neurotrauma. 2020;37(11):1358-1369. 10.1089/neu.2019.6443 31774030 PMC7249471

[5] Zhang W , Hong J, Zhang H, Zheng W, Yang Y. Astrocyte-derived exosomes protect hippocampal neurons after traumatic brain injury by suppressing mitochondrial oxidative stress and apoptosis. Aging. 2021;13(17):21642-21658. 10.18632/aging.203508 34516406 PMC8457605

[6] Meyfroidt G , Bouzat P, Casaer MP, et al. Management of moderate to severe traumatic brain injury: an update for the intensivist. Intensive Care Med. 2022;48(6):649-666. 10.1007/s00134-022-06702-4 35595999

[7] Laskowitz D , Grant G, eds. Translational Research in Traumatic Brain Injury. Boca Raton (FL): CRC Press/Taylor and Francis Group; 2016. 26583170

[8] Akamatsu Y , Hanafy KA. Cell death and recovery in traumatic brain injury. Neurotherapeutics. 2020;17(2):446-456. 10.1007/s13311-020-00840-7 32056100 PMC7283441

[9] Zeng Z , Zhang Y, Jiang W, He L, Qu H. Modulation of autophagy in traumatic brain injury. J Cell Physiol. 2020;235(3):1973-1985. 10.1002/jcp.29173 31512236

[10] Brooks JC , Shavelle RM, Strauss DJ, Hammond FM, Harrison-Felix CL. Long-term survival after traumatic brain injury part II: life expectancy. Arch Phys Med Rehabil. 2015;96(6):1000-1005. 10.1016/j.apmr.2015.02.002 26043195

[11] Brett BL , Gardner RC, Godbout J, Dams-O'Connor K, Keene CD. Traumatic brain injury and risk of neurodegenerative disorder. Biol Psychiatry. 2022;91(5):498-507. 10.1016/j.biopsych.2021.05.025 34364650 PMC8636548

[12] Howlett JR , Nelson LD, Stein MB. Mental health consequences of traumatic brain injury. Biol Psychiatry. 2022;91(5):413-420. 10.1016/j.biopsych.2021.09.024 34893317 PMC8849136

[13] Loane DJ , Faden AI. Neuroprotection for traumatic brain injury: translational challenges and emerging therapeutic strategies. Trends Pharmacol Sci. 2010;31(12):596-604. 10.1016/j.tips.2010.09.005 21035878 PMC2999630

[14] Stelmashook EV , Isaev NK, Genrikhs EE, Novikova SV. Mitochondria-targeted antioxidants as potential therapy for the treatment of traumatic brain injury. Antioxidants. 2019;8(5):124. 10.3390/antiox8050124 31071926 PMC6562849

[15] Cooper DJ , Rosenfeld JV, Murray L, et al; DECRA Trial Investigators. Decompressive craniectomy in diffuse traumatic brain injury [published correction appears in N Engl J Med. 2011 Nov 24;365(21):2040]. N Engl J Med. 2011;364(16):1493-1502. 10.1056/NEJMoa1102077 21434843

[16] Sies H. Oxidative stress: a concept in redox biology and medicine. Redox Biol. 2015;4:180-183. 10.1016/j.redox.2015.01.002 25588755 PMC4309861

[17] Chio JCT , Punjani N, Hejrati N, et al. Extracellular matrix and oxidative stress following traumatic spinal cord injury: physiological and pathophysiological roles and opportunities for therapeutic intervention. Antioxid Redox Signal. 2022;37(1-3):184-207. 10.1089/ars.2021.0120 34465134

[18] Gilkerson R. A Disturbance in the force: cellular stress sensing by the mitochondrial network. Antioxidants. 2018;7(10):126. 10.3390/antiox7100126 30249006 PMC6211095

[19] Oswald MCW , Garnham N, Sweeney ST, Landgraf M. Regulation of neuronal development and function by ROS. FEBS Lett. 2018;592(5):679-691. 10.1002/1873-3468.12972 29323696 PMC5888200

[20] Koch RE , Josefson CC, Hill GE. Mitochondrial function, ornamentation, and immunocompetence. Biol Rev Camb Philos Soc. 2017;92(3):1459-1474. 10.1111/brv.12291 27455896

[21] Long X , Yao X, Jiang Q, et al. Astrocyte-derived exosomes enriched with miR-873a-5p inhibit neuroinflammation via microglia phenotype modulation after traumatic brain injury. J Neuroinflammation. 2020;17(1):89. 10.1186/s12974-020-01761-0 32192523 PMC7082961

[22] Zheng RZ , Lee KY, Qi ZX, et al. Neuroinflammation following traumatic brain injury: take it seriously or not. Front Immunol. 2022;13:855701. 10.3389/fimmu.2022.855701 35392083 PMC8981520

[23] Faden AI , Wu J, Stoica BA, Loane DJ. Progressive inflammation-mediated neurodegeneration after traumatic brain or spinal cord injury. Br J Pharmacol. 2016;173(4):681-691. 10.1111/bph.13179 25939377 PMC4742301

[24] Loane DJ , Kumar A. Microglia in the TBI brain: the good, the bad, and the dysregulated. Exp Neurol. 2016;275(3):316-327. 26342753 10.1016/j.expneurol.2015.08.018 PMC4689601

[25] Shao F , Wang X, Wu H, Wu Q, Zhang J. Microglia and neuroinflammation: crucial pathological mechanisms in traumatic brain injury-induced neurodegeneration. Front Aging Neurosci. 2022;14:825086. 10.3389/fnagi.2022.825086 35401152 PMC8990307

[26] Upadhya R , Madhu LN, Attaluri S, et al. Extracellular vesicles from human iPSC-derived neural stem cells: miRNA and protein signatures, and anti-inflammatory and neurogenic properties. J Extracell Vesicles. 2020a;9(1):1809064. 32944193 10.1080/20013078.2020.1809064 PMC7480597

[27] Upadhya R , Zingg W, Shetty S, Shetty AK. Astrocyte-derived extracellular vesicles: neuroreparative properties and role in the pathogenesis of neurodegenerative disorders. J Control Release. 2020b;323:225-239. 10.1016/j.jconrel.2020.04.017 32289328 PMC7299747

[28] Théry C , Zitvogel L, Amigorena SE. composition, biogenesis and function. Nat Rev Immunol. 2002;2(8):569-579. 12154376 10.1038/nri855

[29] Kim DK , Nishida H, An SY, et al. Chromatographically isolated CD63+CD81+ extracellular vesicles from mesenchymal stromal cells rescue cognitive impairments after TBI. Proc Natl Acad Sci USA. 2016;113(1):170-175. 10.1073/pnas.1522297113 26699510 PMC4711859

[30] Long Q , Upadhya D, Hattiangady B, et al. Intranasal MSC-derived A1-exosomes ease inflammation, and prevent abnormal ­neurogenesis and memory dysfunction after status epilepticus. Proc Natl Acad Sci USA. 2017;114(17):E3536-E3545. 10.1073/pnas.1703920114 28396435 PMC5410779

[31] Upadhya D , Shetty AK. Promise of extracellular vesicles for diagnosis and treatment of epilepsy. Epilepsy Behav. 2021a;121(Pt B):106499. 31636006 10.1016/j.yebeh.2019.106499 PMC7165061

[32] Upadhya R , Shetty AK. Extracellular vesicles for the diagnosis and treatment of Parkinson’s disease. Aging Dis. 2021b;12(6):1438-1450. 10.14336/AD.2021.0516 34527420 PMC8407884

[33] Kalluri R , LeBleu VS. The biology, function, and biomedical applications of exosomes. Science. 2020;367(6478):eaau6977. 32029601 10.1126/science.aau6977 PMC7717626

[34] Denzer K , Kleijmeer MJ, Heijnen HF, Stoorvogel W, Geuze HJ. Exosome: from internal vesicle of the multivesicular body to intercellular signaling device. J Cell Sci. 2000;113(Pt 19):3365-3374. 10.1242/jcs.113.19.3365 10984428

[35] Ma K , Xu H, Zhang J, et al. Insulin-like growth factor-1 enhances neuroprotective effects of neural stem cell exosomes after spinal cord injury via an miR-219a-2-3p/YY1 mechanism. Aging. 2019;11(24):12278-12294. 10.18632/aging.102568 31848325 PMC6949049

[36] Hofer HR , Tuan RS. Secreted trophic factors of mesenchymal stem cells support neurovascular and musculoskeletal therapies. Stem Cell Res Ther. 2016;7(1):131. 10.1186/s13287-016-0394-0 27612948 PMC5016979

[37] Colombo M , Raposo G, Théry C. Biogenesis, secretion, and intercellular interactions of exosomes and other extracellular vesicles. Annu Rev Cell Dev Biol. 2014;30:255-289. 10.1146/annurev-cellbio-101512-122326 25288114

[38] Sonali , Singh RP, Sharma G, et al. RGD-TPGS decorated theranostic liposomes for brain targeted delivery. Colloids Surf B Biointerfaces. 2016;147:129-141. 27497076 10.1016/j.colsurfb.2016.07.058

[39] Zhang L , Liu H, Jia L, et al. Exosomes mediate hippocampal and cortical neuronal injury induced by hepatic ischemia-reperfusion injury through activating pyroptosis in rats. Oxid Med Cell Longev. 2019;2019:3753485. 10.1155/2019/3753485 31814872 PMC6878784

[40] Han Y , Chu X, Cui L, et al. Neuronal mitochondria-targeted therapy for Alzheimer’s disease by systemic delivery of resveratrol using dual-modified novel biomimetic nanosystems. Drug Deliv. 2020;27(1):502-518. 10.1080/10717544.2020.1745328 32228100 PMC7170363

[41] Shi M , Sheng L, Stewart T, Zabetian CP, Zhang J. New windows into the brain: central nervous system-derived extracellular vesicles in blood. Prog Neurobiol. 2019;175:96-106. 10.1016/j.pneurobio.2019.01.005 30685501 PMC6546433

[42] Soares Martins T , Trindade D, Vaz M, et al. Diagnostic and therapeutic potential of exosomes in Alzheimer’s disease. J Neurochem. 2021;156(2):162-181. 10.1111/jnc.15112 32618370

[43] Vogel A , Upadhya R, Shetty AK. Neural stem cell derived extracellular vesicles: attributes and prospects for treating neurodegenerative disorders. EBioMedicine. 2018;38:273-282. 30472088 10.1016/j.ebiom.2018.11.026 PMC6306394

[44] Reis C , Gospodarev V, Reis H, et al. Traumatic brain injury and stem cell: pathophysiology and update on recent treatment modalities. Stem Cells Int. 2017;2017:6392592. 10.1155/2017/6392592 28852409 PMC5568618

[45] Baraniak PR , McDevitt TC. Stem cell paracrine actions and tissue regeneration. Regen Med. 2010;5(1):121-143. 10.2217/rme.09.74 20017699 PMC2833273

[46] Shetty AK. Progress in cell grafting therapy for temporal lobe epilepsy. Neurotherapeutics. 2011;8(4):721-735. 10.1007/s13311-011-0064-y 21892793 PMC3250288

[47] Shetty AK. Hippocampal injury-induced cognitive and mood dysfunction, altered neurogenesis, and epilepsy: can early neural stem cell grafting intervention provide protection? Epilepsy Behav. 2014;38:117-124. 10.1016/j.yebeh.2013.12.001 24433836 PMC4742318

[48] Hattiangady B , Kuruba R, Shuai B, Grier R, Shetty AK. Hippocampal neural stem cell grafting after status epilepticus alleviates chronic epilepsy and abnormal plasticity, and maintains better memory and mood function. Aging Dis. 2020;11(6):1374-1394. 10.14336/AD.2020.1020 33269095 PMC7673840

[49] Lai CP , Breakefield XO. Role of exosomes/microvesicles in the nervous system and use in emerging therapies. Front Physiol. 2012;3:228. 10.3389/fphys.2012.00228 22754538 PMC3384085

[50] Sofroniew MV. Astrocyte barriers to neurotoxic inflammation [published correction appears in Nat Rev Neurosci. 2015 Jun;16(6):372]. Nat Rev Neurosci. 2015;16(5):249-263. 10.1038/nrn3898 25891508 PMC5253239

[51] Burda JE , Bernstein AM, Sofroniew MV. Astrocyte roles in traumatic brain injury. Exp Neurol. 2016;275(3):305-315. 25828533 10.1016/j.expneurol.2015.03.020 PMC4586307

[52] He B , Chen W, Zeng J, Tong W, Zheng P. Long noncoding RNA NKILA transferred by astrocyte-derived extracellular vesicles protects against neuronal injury by upregulating NLRX1 through binding to mir-195 in traumatic brain injury. Aging. 2021;13(6):8127-8145. 10.18632/aging.202618 33686956 PMC8034961

[53] Proia P , Schiera G, Mineo M, et al. Astrocytes shed extracellular vesicles that contain fibroblast growth factor-2 and vascular endothelial growth factor. Int J Mol Med. 2008;21(1):63-67. 18097617

[54] Wang S , Cesca F, Loers G, et al. Synapsin I is an oligomannose-carrying glycoprotein, acts as an oligomannose-binding lectin, and promotes neurite outgrowth and neuronal survival when released via glia-derived exosomes. J Neurosci. 2011;31(20):7275-7290. 10.1523/JNEUROSCI.6476-10.2011 21593312 PMC6622588

[55] Gosselin RD , Meylan P, Decosterd I. Extracellular microvesicles from astrocytes contain functional glutamate transporters: regulation by protein kinase C and cell activation. Front Cell Neurosci. 2013;7:251. 10.3389/fncel.2013.00251 24368897 PMC3857901

[56] Chivet M , Hemming F, Pernet-Gallay K, Fraboulet S, Sadoul R. Emerging role of neuronal exosomes in the central nervous system. Front Physiol. 2012;3:145. 10.3389/fphys.2012.00145 22654762 PMC3361079

[57] Lafourcade C , Ramírez JP, Luarte A, et al. MiRNAs in Astrocyte-derived exosomes as possible mediators of neuronal plasticity. J Exp Neurosci. 2016;10(Suppl. 1):1-9. Published 2016 Aug 8. 10.4137/JEN.S39916 PMC4978198 27547038

[58] Zhao C , Deng Y, He Y, et al. Decreased level of exosomal miR-5121 released from microglia suppresses neurite outgrowth and synapse recovery of neurons following traumatic brain injury. Neurotherapeutics. 2021;18(2):1273-1294. 10.1007/s13311-020-00999-z 33475953 PMC8423926

[59] Ceccarelli L , Giacomelli C, Marchetti L, Martini C. Microglia extracellular vesicles: focus on molecular composition and biological function. Biochem Soc Trans. 2021;49(4):1779-1790. 10.1042/BST20210202 34415305

[60] Van den Broek B , Pintelon I, Hamad I, et al. Microglial derived extracellular vesicles activate autophagy and mediate multi-target signaling to maintain cellular homeostasis. J Extracell Vesicles. 2020;10(1):e12022. 10.1002/jev2.12022 33708355 PMC7890546

[61] Li D , Huang S, Yin Z, et al. Increases in miR-124-3p in microglial exosomes confer neuroprotective effects by targeting fip200-mediated neuronal autophagy following traumatic brain injury. Neurochem Res. 2019;44(8):1903-1923. 10.1007/s11064-019-02825-1 31190315

[62] Murphy DE , de Jong OG, Brouwer M, et al. Extracellular vesicle-based therapeutics: natural versus engineered targeting and trafficking. Exp Mol Med. 2019;51(3):1-12. 10.1038/s12276-019-0223-5 PMC6418170 30872574

[63] Upadhya D , Shetty AK. Extracellular vesicles as therapeutics for brain injury and disease. Curr Pharm Des. 2019;25(33):3500-3505. 10.2174/1381612825666191014164950 31612823

[64] Shahjin F , Chand S, Yelamanchili SV. Extracellular vesicles as drug delivery vehicles to the central nervous system. J Neuroimmune Pharmacol. 2020;15(3):443-458. 10.1007/s11481-019-09875-w 31485884

[65] Fan Y , Li Y, Huang S, et al. Resveratrol-primed exosomes strongly promote the recovery of motor function in SCI rats by activating autophagy and inhibiting apoptosis via the PI3K signaling pathway. Neurosci Lett. 2020;736:135262. 10.1016/j.neulet.2020.135262 32682847

[66] Liu X , He F, Pang R, et al. Interleukin-17 (IL-17)-induced microRNA 873 (miR-873) contributes to the pathogenesis of experimental autoimmune encephalomyelitis by targeting A20 ubiquitin-editing enzyme. J Biol Chem. 2014;289(42):28971-28986. 10.1074/jbc.M114.577429 25183005 PMC4200254

[67] Sarkar C , Zhao Z, Aungst S, et al. Impaired autophagy flux is associated with neuronal cell death after traumatic brain injury. Autophagy. 2014;10:2208-2222. 10.4161/15548627.2014.981787 25484084 PMC4502690

[68] Sun L , Zhao M, Wang Y, et al. Neuroprotective effects of miR-27a against traumatic brain injury via suppressing FoxO3a-mediated neuronal autophagy. Biochem Biophys Res Commun. 2017;482:1141-1147. 10.1016/j.bbrc.2016.12.001 27919684

[69] Stocchetti N , Zanier ER. Chronic impact of traumatic brain injury on outcome and quality of life: a narrative review. Crit Care. 2016;20(1):148. 10.1186/s13054-016-1318-1 27323708 PMC4915181

[70] Manley G , Gardner AJ, Schneider KJ, et al. A systematic review of potential long-term effects of sport-related concussion. Br J Sports Med. 2017;51(12):969-977. 10.1136/bjsports-2017-097791 28455362 PMC5466926

[71] LoBue C , Munro C, Schaffert J, et al. Traumatic brain ­injury and risk of long-term brain changes, accumulation of patho­logical markers, and developing dementia: a review. J Alzheimers Dis. 2019;70(3):629-654. 10.3233/JAD-190028 31282414

[72] Kodali M , Castro OW, Kim DK, et al. Intranasally administered human msc-derived extracellular vesicles pervasively incorporate into neurons and microglia in both intact and status epilepticus injured forebrain. Int J Mol Sci. 2019;21(1):181. 10.3390/ijms21010181 31888012 PMC6981466

